# Socio-cognitive factors influencing access to HIV prevention services among people who inject drugs in Dar es Salaam, Tanzania: An integrated bio-behavioural survey

**DOI:** 10.1371/journal.pone.0261500

**Published:** 2022-01-28

**Authors:** Samuel L. Likindikoki, Dan W. Meyrowitsch, Mucho M. Mizinduko, Alexander M. Ishungisa, Britt P. Tersbøl, Germana H. Leyna, Kåre Moen, Neema Makyao, Theis Lange, Melkizedeck T. Leshabari, Elia J. Mmbaga

**Affiliations:** 1 Department of Epidemiology and Biostatistics, Muhimbili University of Health and Allied Sciences, Dar es Salaam, Tanzania; 2 Department of Psychiatry and Mental Health, Muhimbili University of Health and Allied Sciences, Dar es Salaam, Tanzania; 3 Department of Public Health, Global Health Section, University of Copenhagen, Copenhagen, Denmark; 4 Department of Behavioural Sciences, Muhimbili University of Health and Allied Sciences, Dar es Salaam, Tanzania; 5 Tanzania Food and Nutrition Centre, Dar es Salaam, Tanzania; 6 Department of Community Medicine and Global Health, Institute of Health and Society, University of Oslo, Oslo, Norway; 7 National AIDS Control Programme, Ministry of Health, Community Development, Gender, Children and Elderly, Dodoma, Tanzania; 8 Department of Public Health, Section of Biostatistics, University of Copenhagen, Copenhagen, Denmark; University of Washington, UNITED STATES

## Abstract

**Introduction:**

People who inject drugs (PWID) in Sub-Saharan Africa have limited access to comprehensive HIV services. While it is important to inform programming, knowledge about factors influencing access to comprehensive HIV services is scarce. We assessed the proportions of PWID with access to HIV prevention services and associated socio-cognitive factors in Tanzania.

**Methods:**

A cross-sectional survey was conducted among PWID between October and December 2017 in Dar es Salaam, Tanzania. Data on access to HIV prevention services, demographics and selected socio-cognitive factors were collected through structured face-to-face interviews. Weighted descriptive and forward selection multivariable logistics regression analyses were done to assess independent associations between HIV prevention services and predictors of interest. The results were two tailed and a *p-value* of less than 0.05 was considered statistically significant.

**Results:**

The study included 611 PWID (males: 94.4%) with a median age of 34 years (Interquartile Range (IQR), 29–38). A large majority of participants reported to have access to condoms (87.8%), sterile needles/syringes (72.8%) and ever tested for HIV (66.0%). About half (52.0%) reported to have used condoms in the past one month and about a third (28.5%) accessed a peer educator. The odds of testing for HIV decreased among participants who perceived their HIV risk to be high (aOR = 0.29; 95%CI: 0.17–0.49) and those experienced sexual violence (aOR = 0.60; 95%CI 0.37–0.98). However, the odds of testing for HIV increased among participants with secondary level of education (aOR = 2.16; 95%CI: 1.06–5.55), and those who reported having correct comprehensive HIV knowledge (CCHK) (aOR = 1.63; 95%CI 1.12–2.41). The odds of access to condoms increased among females (aOR = 2.23; 95%CI: 1.04–5.02) but decreased among participants with secondary level of education (aOR = 0.41; 95%CI: 0.19–0.84), an income of >TZS 200,000 (aOR = 0.39; 95%CI: 0.23–0.66) and those who perceived their HIV risk to be high (aOR = 0.13; 95%CI: 0.03–0.36). The odds of access to peer educators was higher among participants with primary (aOR = 1.61; 95%CI: 1.01–2.26), and secondary (aOR = 2.71; 95%CI: 1.39–5.33) levels of education. The odds of access to sterile needle and syringe decreased among participants who perceived their HIV risk to be high (aOR = 0.11;95%CI 0.05–0.22), and low-medium (aOR = 0.25;95%CI 0.11–0.52) but increased among those with primary level of education (aOR = 1.72;95%CI 1.06–2.78).

**Conclusion:**

Access to condom, HIV testing, sterile needles and syringes were relatively high among PWID. However, condom use and access to peer educators was relatively low. HIV knowledge and risk perception, gender, education, and sexual violence influenced access to HIV prevention services. There is an urgent need to address the identified socio-cognitive factors and scale up all aspects of HIV prevention services to fast-track attainment of the 2025 UNAIDS goals and ending the HIV epidemic.

## Introduction

Injection drug use is a public health concern and is associated with lost productivity and increased health care expenses [1, 2]. Globally, it is estimated that there are about 16 million people who inject drugs (PWID) aged 15 to 64 years and that 17.8% of them are living with HIV [3]. Recent data suggests that injection drug use contributes between 10% and 15% of all new HIV infections [4] and hamper actions to contain the global HIV epidemic [5]. Injection drug use is also associated with negative social [6] and health consequences such as infections [7–11] and mental health problems like depression [12], personality disorders [13], and suicidal behaviour [7, 14].

Optimal access to comprehensive HIV services among PWID is one of the key strategies to reduce HIV infection among PWID [15]. Despite this, availability of and access to HIV prevention interventions is still limited in most countries [16], including Tanzania. UNAIDS set ambitious targets for mitigation of HIV and AIDS by 2020, aiming to have 90% of people living with HIV know their status; and 90% of these should receive antiretroviral therapy (ART) of which 90% should be virally suppressed [17]. In addition, UNAIDS set specific targets for PWID that by 2025, 90% should have access to clean needles and syringes, 75% should have contact with harm reduction services, 95% of HIV positive PWID should be enrolled into opioid substitution therapy (OST) and ART, and 40% should receive adequate OST dosage [18]. However, achieving these targets faces many challenges including stigma, discrimination and social exclusion [19–23].

In 2014, Tanzania initiated the implementation of the National Guideline for Comprehensive Package of HIV Interventions for Key Populations. The package contains items related to HIV prevention for PWID such as provision of OST, HIV testing and counselling, ART, screening for viral hepatitis and tuberculosis (TB), condom programming and health information [24]. Studies suggest that factors such as inadequate social support and stability, stigma, violence, knowledge about HIV, attitudes towards healthcare providers, and perceived risk for HIV play a major role in the access to different components of health services among PWID [25–27]. Since the rollout of the national guidelines for comprehensive HIV services for key and vulnerable populations in Tanzania, there has been no study on the levels of access to the offered HIV prevention services and the role socio-cognitive factors play in influencing access among PWID. Therefore, the present study aims were to 1) assess access to different components of HIV prevention services; and 2) determine the role of socio-cognitive factors in the access to different components of HIV prevention services among PWID.

## Materials and methods

### Study design and setting

A cross-sectional integrated bio-behavioural survey (IBBS) was carried out between October and December 2017 in Dar es Salaam, Tanzania. Dar es Salaam is Tanzania’s largest city with an estimated population of 6 million [28]. Injecting drug use is reported to be widespread in the city since the late 1990’s. It is estimated that Dar es Salaam is home to about 30–50% of the total PWID population in Tanzania [29]. Despite a decreasing trend in the general population, the prevalence of HIV has been reported to be higher among PWID. A recent study reported an HIV prevalence of 8.7% among PWID in Dar es Salaam which is a decline compared to a previous estimate of 15.5% in 2014 [30, 31].

### Sample size

Sample size calculation was based on estimates for the parent study which aimed at examining the prevalence of HIV infection among PWID. Power and sample size were based on the prevalence of HIV (15.5%) among PWID, and the precision was set at 5% [31], which implied a targeted sample size of 610. This sample size is adequate to estimate the proportion of access to services in this population given the relatively high proportion of reported access to most services.

### Study population

The study population was people who inject drugs aged 18 years and older at the time of data collection. Participants were recruited if they had reported to have injected drugs three months prior to recruitment and have been living in Dar es Salaam for the last 6 months prior to the survey.

### Participant’s recruitment and data collection procedures

The details of the sampling procedures for this study have been presented in a previous publication [30]. In summary, respondent-driven sampling (RDS) was used to recruit study participants. RDS is a method used for recruitment from populations for which a sampling frame is impossible to obtain [32].

Five research assistants (RAs) with experience in conducting surveys on HIV among at-risk populations were recruited and trained on; the study protocol, data collection instruments that were uploaded into open data collection kit (ODK), research ethics and sensitivity in working with key and vulnerable populations. The sensitivity training addressed stereotyping and prejudices related to key, vulnerable and marginalized populations.

Investigators in collaboration with Peer Educators and RAs purposefully selected and recruited a total of five “seeds” (i.e., the initial survey participants). Seeds were selected from various strata to reduce the chances that all might be drawn from the same network of PWID. We diversified the representation of seeds based on location within the city, age, sex, income, and education levels. Subsequently, each seed was given a maximum of three coupons to recruit up to three potential participants, and the process continued until the estimated sample size was reached.

A “screener”—a person who had injected drugs in the past, used a standard screening tool to assess all potential study participants for eligibility before they were enrolled into the study. The screener received a two-day training aiming to ensure objectivity in the screening process. Injection track marks, knowledge of local terms related to injection practices, and drug prices were the criteria used to verify eligibility for participation. Possessing a valid recruitment coupon was a prerequisite for inclusion in the study. Each coupon had a unique electronic barcode that linked recruiters to recruitees for network size estimation and the same barcode was also used on consent forms, questionnaires, and laboratory specimens.

Structured face-to-face interviews were carried out in a private, safe location which was easily accessible to the participants. The information gathered was recorded using an ODK. Soon after data had been collected, a Coupon Manager issued three coupons to the recruiter (with the intention that these be used to recruit other PWID into the study). A total of 8,000 Tanzania Shillings (equivalent to US$ 3.50) was paid as transport reimbursement (covering the cost for traveling to the study centre) and 4,000 Tanzanian Shillings (equivalent to US$ 1.75) was paid as recruitment reimbursement (covering the cost for recruiting others into the study). The reimbursements were approved by the Muhimbili University of Health and Allied Sciences (MUHAS) ethics committee.

### Data collection tool

The questionnaire was adapted from previous surveys among PWID in Dar es Salaam [31]. No validation of the tools was done because they had been used in a similar survey in Dar es Salaam three years earlier and yielded robust results [31]. The previous survey tool was based on the instruments employed by the World Health Organization (WHO), and the toolbox for conducting HIV bio-behavioural surveillance among key populations from University of California, San Francisco. A measure of Correct Comprehensive HIV Knowledge included a number of questions that had been used in the Tanzania Demographic and Health Surveys [33]. Pre-testing of tools was done with peer educators to ensure cultural and peer relevance. The questionnaire gathered information on demographics, socio-cognitive factors, and access to HIV prevention services. All interviews were done in Swahili, the official and widely spoken language in Tanzania.

### Measures

The outcome measures for this study were based on self-reported access to five HIV prevention services and included; 1) access to HIV testing services; 2) ability to obtain a male condom every time needed; 3) having used a male condom in the past one month; 4) having ever contacted a peer educator, and 5) access to sterile needles and syringes. Access and usage of HIV prevention services were measured as binary variables. HIV testing was measured by asking; Have you ever had an HIV test? And response was recoded as yes or no. Access to sterile needles and syringes was measured by asking; Can you get a clean needle and syringe any time you need one? And the response was recorded as yes or no. Access to condom was measured by asking; can you obtain a male condom every time you need one? And response was recorded as yes or no. Condom use in the past one month was measured by asking; If you had vaginal or anal sex during the past one month, how often did you use condom? And responses were never recorded as no, occasionally, most of the time and always recorded as yes. The survey tool collected information on various socio-demographic characteristics such as age (continuous), gender (binary), marital status (categorical), income per month (continuous), employment status (categorical) and level of education (categorical). The socio -cognitive factors measured in this survey included; Correct Comprehensive HIV knowledge (CCHK), experienced stigma, perception of HIV risk, and experience of violence (sexual, physical or both). The level of CCHK was measured using elements similar to those used by the Tanzania Demographic and Health Surveys [33] where, CCHK was defined as being able to provide correct answers to at least four of seven questions. The questions were related to knowledge about the protection provided by consistent use of condom, having a single uninfected faithful partner, the use of sterile needles, knowing that a healthy-looking person can have HIV/AIDS and rejecting common misconceptions about HIV/AIDS transmission and prevention (for example that a person can get HIV from mosquito bites, or from sharing food with someone who has HIV). Relevant to the study population, a question on the HIV transmission risk associated with needle sharing was also included. Experienced stigma was measured by asking participants to respond to a set of five statements referring to their own experiences. These statements covered issues related to: 1) name-calling, teasing and insults; 2) having been excluded from a social gathering; 3) having been gossiped about; 4) having lost respect from other people, and 5) having been abandoned. Participants reporting none or one of these five experiences were categorized as having experienced no or a low level of stigma; participants reporting two to three of the five experiences were categorized as having experienced a moderate level of stigma and participants reporting four to five elements were categorized as having experienced a high level of stigma. The perception of own HIV risk was measured by asking participants to respond to five statements related to their perception of own HIV risk: 1) I perceive that I am at risk of HIV because I do not use a condom; 2) I perceive that I am at risk of HIV because I have multiple sexual partners; 3) I perceive that I am at risk of HIV because I share needles and syringes; 4) I perceive that I am at risk of HIV because I inject drugs, and 5) I perceive that I am at risk of HIV because I use drugs. If a participant verified one of these statements, two or three of these statements, or four or all statements, the person was categorized as having a low, medium or a high levels of perceived own HIV risk, respectively. Experience of violence was measured using two questions: 1) In the past 12 months were you ever forced to have sex; and 2) In the past 12 months were you ever beaten?

### Statistical analyses

Data were analysed using the RDS Analysis Tool (RDSAT), STATA version 15.1 and R-Studio. RDSAT calculated weight as an inverse of the participant’s network size and controlled for clustering by multiplying the weight by the sample size and divided by the sum of the weight. The median and interquartile range (IQR) were used to summarize continuous variables. Frequencies and proportions were used to summarize categorical variables. Logistic regression models were used to estimate the associations between socio-cognitive predictors and access to HIV prevention services. Forward selection multivariable logistic regression modelling was used to determine independent determinants of access to different HIV prevention services. The models with the lowest Akaike Information Criterion (AIC) were considered as the final models. Crude odds ratios (cOR) and adjusted odds ratios (aOR) with their corresponding 95% confidence intervals were reported. All tests were two-tailed, and a *p-value* of less than 0.05 was considered statistically significant.

### Ethical considerations

The study was approved by the Muhimbili University of Health and Allied Sciences’ ethical review committee. Participants provided written informed consents before interviews. I addition, the participants received health education on HIV prevention during their visits to the study centre.

## Results

### Characteristics of the study participants

A total of 611 PWID participated in the study and the distribution of their socio-demographic characteristics are shown in Table 1. A large majority of the participants were males (94.0%) and aged between 25 and 44 years (84.0%,) The median age was 34 years (IQR, 29–38). Almost two-thirds (67.6%) of the participants had completed primary school education, whereas about a quarter (23.5%) had no formal education. About half (52.7%) of the participants reported to be single, and three-quarters (74.7%) were self-employed. A third (36.4%) reported earnings of more than 200,000 Tanzania Shillings (equivalent to US$ 87) per month (Table 1).

**Table 1:** Distribution of socio-demographic characteristics of study participants.

Characteristics	Unweighted	Weighted
	n (%)	(%)
**Age**		
< 25	54 (8.8)	8.7
25–34	267 (43.7)	43.0
35–44	246 (40.3)	41.0
≥ 45	44 (7.2)	6.8
**Sex**		
Male	576 (94.3)	94.4
Female	35 (5.7)	5.6
**Education level**		
None	133 (21.8)	23.5
Primary	418 (68.4)	67.6
Secondary	60 (9.8)	8.9
**Marital status**		
Never married	323 (52.9)	52.7
Married partner	115 (18.8)	17.1
Separated/divorced/widowed	173 (28.3)	30.2
**Occupational status**		
None	24 (3.9)	3.6
Self-employed	450 (73.7)	74.7
Employed	100 (16.4)	16.3
informal activities	37 (6.0)	5.4
Income level per month (Tshs)*		
<50,000	116 (19.0)	20.3
50,000–120,000	145 (23.7)	25.7
120,001–200,000	95 (15.6)	17.6
>200,000	255 (41.7)	36.4

*Tshs = Tanzanian Shilling.

### Access to HIV prevention services

Of the 611 PWID who participated in the study, about two-thirds (66.0%) reported to have ever been tested for HIV. A large majority (87.8%) reported that they could obtain condoms whenever they needed, About half (52%) of the participants reported to have used condom in the past one month and nearly one-third (27.2%) reported to have ever contacted a peer educator. Additionally, nearly three-quarters (69.7%) reported having access to sterile needles and syringes (Table 2).

**Table 2:** Access to HIV prevention services among people who inject drugs in Dar es Salaam, Tanzania (n = 611).

Selected Preventive services	Unweighted	Weighted
	n (%)	(%)
**Ever tested for HIV infection**		
Yes	414 (67.8)	66.0
No	197 (32.2)	34.0
**Obtain condom every time needed**		
Yes	406 (89.2)	87.8
No	49 (10.8)	12.2
**Used condom past one month**		
Yes	255 (56.0)	52.0
No	200 (43.0)	48.0
**Contacted a peer educator**		
Yes	174 (28.5)	27.2
No	437 (71.5)	72.8
**Access to sterile needles and syringes**		
Yes	429 (70.2)	69.7
No	182 (29.8)	30.3

### Distribution of socio-cognitive factors

A large proportion of the participants (70.7%) reported to have experienced higher levels of stigma. About 4 out of every 10 participants (38.9%) had correct comprehensive HIV knowledge (CCHK). Additionally, about a third (34.8%) and 14.2% of the participants reported to have experienced physical and sexual violence in the past one month, respectively. Half (53.6%) of participants perceived their level of HIV risk to be high, whereas 19.0% perceived not to be at risk for HIV infection (Table 3).

**Table 3:** Frequency distribution of socio-cognitive factors among people who inject drugs (n = 611).

Socio-cognitive factors	Unweighted	Weighted
	n (%)	(%)
**Experienced stigma**		
Low stigma (scores 0–1)	63 (10.3)	10.2
Moderate stigma (scores 2–3)	109 (17.8)	19.1
High Stigma (4–5)	439 (71.9)	70.7
**Comprehensive Correct HIV Knowledge (CCHK)**		
Yes (scores 4–7)	247 (40.4)	38.9
No (scores 0–3)	364 (59.6)	61.1
**Experienced physical violence in the past 12 months**		
Yes	226 (37.0)	34.8
No	385 (63.0)	65.2
**Experience sexual violence in the past 12 months**		
Yes	91 (14.9)	14.2
No	520 (85.1)	85.8
**Experience physical and or sexual violence in the past 12 months**		
Yes	272 (44.5)	42.2
No	339 (55.5)	57.8
**Perceived HIV risk**		
Low risk (scores 0–1)	128 (20.9)	19.5
Medium risk (scores 2–3)	165 (27.0)	26.9
High risk (scores 4–5)	318 (52.1)	53.6

### Predictors of access to HIV testing services

Bivariate and multivariable analyses of the association between socio-demographics, cognitive factors and access to HIV prevention services are presented in Tables 4 and 5. Results of multivariate analysis of independent predictors of access to health services are further described in detail.

**Table 4:** Unadjusted analysis of the association between socio-demographic, cognitive factors and access to HIV prevention services among people who inject drugs in Dar es Salaam, 2017, (N = 611).

Covariates	HIV prevention services				
	Tested for HIV	Obtain condom	Past month condom use	Peer educator	Sterile needle
	cOR (95% CI)	cOR (95% CI)	cOR (95% CI)	cOR (95% CI)	cOR (95% CI)
**Age**					
<25	1	1	1	1	1
25–34	1.04 (0.55–1.97)	0.07 (0.23–2.12)	0.67 (0.35–1.29)	1.14 (0.59–2.21)	0.95 (0.50–1.82)
35–44	0.76 (0.40–1.43)	0.95 (0.30–2.97)	0.59 (0.31–1.15)	1.11 (0.57–2.18)	0.81 (0.42–1.56)
45+	0.74 (0.32–1.72)	1.00 (0.21–4.81)	0.54 (0.22–1.35)	1.48 (0.62–3.53)	1.15 (0.47–2.85)
**Sex**					
Male	1	1	1	1	1
Female	1.04 (0.50–2.17)	1.88 (0.43–8.10)	2.60 (1.22–5.53) *	0.61 (0.26–1.43)	0.62 (0.31–1.24)
**Education level**					
None	1	1	1	1	1
Primary	1.41 (0.94–2.11)	0.64 (0.28–1.50)	0.61 (0.38–0.98) *	1.62 (1.01–2.60) *	1.49 (0.76–2.07)
Secondary	2.11 (1.06–4.21) *	0.77 (0.23–2.56)	0.38 (0.19–0.79) *	2.80 (1.44–5.45) *	0.21 (0.64–2.33)
**Marital status**					
Never married	1	1	1	1	1
Married partner	0.75 (0.48–1.17)	0.83 (0.37–1.85)	0.84 (0.50–1.42)	0.92 (0.56–1.50)	0.76 (0.48–1.20)
Separated/divorced/widowed	0.85 (0.57–1.26)	0.93 (0.47–1.86)	0.67 (0.43–1.03)	1.59 (1.07–2.37) *	0.78 (0.52–1.17)
**Occupational status**					
Employed	1	1	1	1	1
Self employed	0.54 (0.32–1.81)	0.69 (0.28–1.69)	0.91 (0.55–1.49)	0.77 (0.49–1.22)	0.86 (0.52–1.40)
Informal activities	0.76 (0.32–0.90) *	0.45 (0.13–1.60)	1.80 (0.78–4.14)	0.54 (0.22–1.30)	0.37 (0.17–0.81) *
None	0.39 (0.15–1.01) *	0.53 (0.12–2.31)	0.85 (0.33–2.22)	0.39 (0.12–1.23)	0.85 (0.32–2.29)
Income level per month (Tshs)*					
<50,000	1	1	1	1	1
50,000–120,000	0.82 (0.49–1.39)	0.70 (0.27–1.78)	0.80 (0.44–1.44)	0.83 (0.48–1.44)	1.38 (0.81–2.34)
120,001–200,000	0.811 (0.45–1.45)	1.95 (0.56–0.75)	0.75 (0.40–1.39)	1.05 (0.58–1.91)	1.21 (0.68–2.18)
>200,000	0.96 (0.60–1.55)	0.77 (0.33–1.79)	0.38 (0.22–0.64) *	1.09 (0.67–1.77)	1.19 (0.74–1.91)
**Experienced stigma**					
Low stigma (scores 0–1)	1	1	1	1	1
Moderate stigma (scores 2–3)	0.94 (0.46–1.91)	0.77 (0.14–4.15)	0.86 (0.40–1.82)	1.39 (0.66–2.93)	0.60 (0.27–1.35)
High Stigma (4–5)	0.64 (0.35–1.17)	0.36 (0.08–1.53)	0.91 (0.48–1.75)	1.65 (0.87–3.15)	0.38 (0.19–0.77) *
**CCHK**					
No (scores 0–3)	1	1	1	1	1
Yes (scores 4–7)	1.68 (1.16–2.41) *	1.44 (0.77–2.70)	0.98 (0.67–1.44)	1.07 (0.74–1.54)	1.12 (0.80–1.65)
**Experience of sexual violence**					
No	1	1	1	1	1
Yes	0.55 (0.35–0.87) *	0.54 (0.26–1.12)	1.17 (1.05–2.99) *	1.36 (0.85–2.19)	0.37 (0.23–0.58) *
**Experience of physical violence**					
No	1	1	1	1	1
Yes	1.00 (0.70–0.87) *	0.89 (0.48–1.62)	0.84 (0.58–1.24)	0.95 (0.66–1.37)	0.98 (0.68–1.40)
**Experience of physical and /or sexual violence**					
No	1	1	1	1	1
Yes	0.88 (0.62–1.23)	0.76 (0.42–1.37)	0.91 (0.63–1.32)	1.24 (0.87–1.76)	0.80 (0.57–1.14)
**Perception of own HIV risk**					
No-Low risk (scores 0–1)	1	1	1	1	1
Medium risk (scores 2–3)	0.48 (0.27–0.85) *	0.70 (0.16–3.01)	1.32 (0.77–2.25)	0.76 (0.46–1.25)	0.24 (0.11–0.51)*
High risk (scores 4–5)	0.29 (0.17–0.49) *	0.13 (0.04–0.45)*	1.59 (0.99–2.56)	0.67 (0.43–1.05)	0.11 (0.05–0.22) *

CCHK = Comprehensive Correct HIV knowledge; cOR = Crude odds ratio

*p-value <0.05; CI = Confidence Interval.

**Table 5:** Multivariable logistic regression of association between socio-demographic, cognitive factors and access to HIV prevention services among people who inject drugs in Dar es Salaam, 2017, (N = 611).

Covariates	Access to HIV prevention services				
	HIV testing	Obtain condom	Use condom past month	Peer educator	Sterile needle
	aOR (95% CI)	aOR (95% CI)	aOR (95% CI)	aOR (95% CI)	aOR (95% CI)
**Sex**					
Male	*NA*	*Ref*	*Ref*	*NA*	*NA*
Female	*NA*	3.01 (0.85–19.14)	2.23 (1.04–5.02) *	*NA*	*NA*
**Education**					
None	*Ref*	*NA*	*Ref*	*Ref*	*Ref*
Primary	1.37 (0.89–2.10)	*NA*	0.61 (0.37–1.00)	1.61 (1.01–2.26) *	1.72 (1.06–2.78) *
Secondary	2.16 (1.06–4.55) *	*NA*	0.41 (0.19–0.84) *	2.71 (1.39–5.33)	1.62 (0.77–3.50)
**Marital status**					
Never married	*NA*	*NA*	*NA*	*Ref*	*Ref*
Married partner	*NA*	*NA*	*NA*	0.86 (0.52–1.40)	0.63 (0.40–1.04)
Separated/divorced/widowed	*NA*	*NA*	*NA*	1.51 (1.01–2.52) *	0.73 (0.48–1.13)
**Occupational status**					
Employed	*Ref*	*NA*	*NA*	*NA*	*NA*
Self employed	0.55 (0.32–0.93) *	*NA*	*NA*	*NA*	*NA*
None	0.47 (0.18–1.26)	*NA*	*NA*	*NA*	*NA*
Informal activities	1.17 (0.47–3.02)	*NA*	*NA*	*NA*	*NA*
**Income level per month (Tshs)**					
<50,000	*NA*	*NA*	*Ref*	*NA*	*NA*
50,000–120,000	*NA*	*NA*	0.85 (0.47–1.55)	*NA*	*NA*
120,001–200,000	*NA*	*NA*	0.74 (0.39–1.38)	*NA*	*NA*
>200,000	*NA*		0.39 (0.23–0.66) *	*NA*	*NA*
**CCHK**					
No (scores 0–3)	*Ref*	*NA*	*NA*	*NA*	*NA*
Yes (scores 4–7)	1.63 (1.12–2.41) *	*NA*	*NA*	*NA*	*NA*
**Sexual violence**					
No	*Ref*	*NA*	*NA*	*NA*	*Ref*
Yes	0.60 (0.37–0.98) *	*NA*	*NA*	*NA*	0.50 (0.31–0.81) *
**Perception of own HIV risk**					
No (scores 0–1)	*Ref*	*Ref*	*NA*	*NA*	*Ref*
Low—Medium risk (scores 2–3)	0.50 (0.27–0.89) *	0.69 (0.14–2.87)	*NA*	*NA*	0.25 (0.11–0.52) *
High risk (scores 4–5)	0.29 (0.17–0.49) *	0.13 (0.03–0.36) *	*NA*	*NA*	0.11 (0.05–0.22) *

CCHK = Correct Comprehensive HIV knowledge; aOR = adjusted odds ratio

’*’ *p-value <*0.05; CI = Confidence Interval; Ref: Reference group Tshs = Tanzanian Shillings.

Forward selection multivariable logistic regression analyses was used to evaluate independent association between sociodemographic factors, cognitive factors and access to HIV prevention services. The findings of the final regression model indicated that the level of education, occupation status, having correct comprehensive HIV knowledge, experience of sexual violence and perception of own HIV risk independently predicted access to HIV testing. Participants who reported having secondary level of education and above (aOR = 2.16; 95%CI 1.06–4.55) had twice higher odds of testing for HIV than those who reported not having formal education. Participants who reported being self-employed (aOR = 0.55; 95% CI 0.32–0.93) and experienced sexual violence (aOR = 0.60; 95%CI 0.37–0.98) had decreased odds of testing for HIV than those who reported to be employed and not experienced sexual violence, respectively. Participants with correct comprehensive HIV knowledge (aOR = 1.63; 95%CI 1.12–2.41) were more likely to report having tested for HIV than those with no correct comprehensive HIV knowledge. Moreover, participants who perceived their own HIV risk to be high (aOR = 0.29; 95% CI 0.17–0.49) and those who perceived it to be medium (aOR = 0.50; 95%CI 0.27–0.89) had decreased odds of having tested for HIV than those who perceived their HIV risk to be low or none. However, age, gender, marital status, income, experiences of stigma and physical violence were not found to be independently associated with access to HIV testing (Table 5).

### Predictors of condom accessibility and use

Participants who perceived their HIV risk to be high (aOR = 0.13; 95% CI 0.03–0.36) had decreased odds of obtaining a condom than those who perceived their own HIV risk to be low or none. With regards to past month condom use; participants who reported having secondary level of education and above (aOR = 0.41; 95% CI; 0.19–0.84) and those with income of > TShs. 200,000 had decreases odds of reporting condom use in the past one month preceding the survey than those with no formal education and income of < TShs. 50,000, respectively. However, being a female PWID (aOR = 2.23; 95% CI 1.04–5.02) was independently associated with higher odds of reporting condoms use in the past one month (Table 5).

### Predictors of access to peer educators’ services

Multivariable logistic regression analyses (Table 5) indicated that, participants with primary (aOR = 1.61; 95% CI 1.01–2.26) and secondary levels of education (aOR = 2.71; 95% CI 1.39–5.33) had increased odds of access to a peer educator than those with no formal education. Moreover, participants who reported being either separated, divorced or widowed (aOR = 1.51; 95% CI; 1.01–2.52), had higher odds of access to peer educator than those who reported to have never being married.

#### Predictors of access to sterile needles and syringes

Participants who reported to have completed primary level of education (aOR = 1.72; 95% CI 1.06–2.78) had increased odds of access to sterile needles and syringes than those with no formal education. Participants who reported to have experienced sexual violence in the past one month (aOR = 0.50; 95% CI; 0.31–0.81) and those having a high perceived own HIV risk (aOR = 0.11; 95% CI; 0.05–0.22) and a medium perceived own HIV risk (aOR = 0.25; 95% CI; 0.11–0.52) had decreased odds of access to sterile needles and syringes (Table 5).

## Discussion

Given the risks of HIV acquisition and transmission associated with injecting drug use, access to HIV prevention services is of paramount importance. Our study aimed to evaluate the proportions of access to HIV prevention services and determine the social and cognitive factors influencing such access. To our knowledge, this is the first study that has documented access to several elements of HIV prevention services among PWID in Tanzania. We found relatively high proportions of PWID reporting access to HIV testing, sterile needles, and syringes. However, the proportions of condom use in the past one month, and access to peer educators = were relatively low. Not all socio-demographic and socio-cognitive factors were significantly influencing access to HIV preventive services. Gender, level of education, correct comprehensive HIV knowledge, perception of HIV own risk, and sexual violence were found to independently influence access to different elements of HIV prevention services.

Our study found that about two-thirds of PWID had ever tested for HIV, which is similar to that of the adult general population in Tanzania [33]. However, our findings on HIV testing reflected on lifetime ever tested experience and not whether they had recently tested for HIV. While most African countries do not have reliable data on access to HIV testing among PWID, the few data that are available indicate that access to HIV testing among PWID ranges from 11% to 100% [34]. In 2013, Terlikbayeva et al. reported a similar proportion of HIV testing among PWID in Central Asia [35]. However, studies in North America and Europe depart from our findings as they show a significant stride in HIV testing among PWID that surpasses the UNAIDS target [36–38]. HIV testing and counselling are among the key strategies for ending HIV and AIDS and is one of the necessary steps in the prevention of HIV infection [39, 40]. The proportion of HIV testing for PWID in this study is promising despite being lower than the anticipated first UNAIDS 90 goal. While the proportion of HIV testing reported in our study indicate progress towards a positive direction, it is critical for HIV programmes in Tanzania to target more PWID, increase the proportion of those who receive HIV testing, to link those who are positive to HIV care, and to make sure that those who receive ART are virally suppressed to be able to significantly curb the HIV transmission.

Access to sterile needles and syringes is critical in reducing HIV transmission among PWID [41, 42]. Our study found that a majority of participants had access to sterile needles and syringes. Tanzania is one of the countries in Sub-Saharan Africa that piloted the needle and syringe programming [43]. These findings indicate success of the ongoing harm reduction interventions in the country. However, there is a need to step up and expedite the needle and syringe exchange programming to reach the remaining segments of PWID and to be able to realize the UNAIDS global targets.

Access to a condom (87.8%) was high in this population with about a half reporting to have used a condom in the past one month (52%.). While the levels of access to condoms among PWID in our study was relatively high, the proportion of those who recently used a condom is relatively low. Comparable to our findings, Marshal et al. in Canada reported considerably lower proportion of condom use among PWID either with regular or casual partners [44]. Furthermore, we found that females were more likely to use condoms than males. The observed gender difference in condom use is consistent with a previous study done in Tanzania that showed women who inject drugs were more likely to have used a condom with the most recent sex partner than their male counterpart [45]. This could be because women who inject drugs have extra layers of HIV risks related to sex work and hence may also be reached by condom programming targeting female sex workers [46]. Our findings underscore the need to continue to address both condom accessibility and use among PWID. For instance, more advocacy for health promotion interventions focusing on increased access to condom and ensure that people who use drugs have adequate knowledge on correct comprehensive use of condoms.

Demographic factors such as gender, level of education and marital status were found to be associated with access to different components of HIV prevention services among people who inject drugs. Our study revealed that participants’ level of education had influence on access to different elements of HIV prevention services. Having primary or secondary education was positively associated with; HIV testing, condom use in the past one month, access to peer educator and sterile needles and syringes. With the exception of reported use condom in the past one month, our study found that gender was not an independent predictor of access to other HIV prevention services, i.e. access to condom, sterile needles and syringes, and peer educator’s services. Marital status is one of the demographic factors found to influence access to peer educators. Participants who are either divorced, separated or widowed were more likely to contact peer educators than those who were single. Not surprisingly, peer educators perhaps could be providing alternative social safety-net for people who inject drugs who are not enjoying the support from primary partners.

We found that experience of sexual violence was a deterrent factor in accessing both HIV testing and sterile needle and syringes. Other studies corroborate findings from this study [47, 48].

About half of the participants in this study perceived themselves to be at higher risk of HIV infection, whereas about 1 in 5 participants perceived to be at low or no risk. Participants with heightened perception of own HIV risk were less likely to; test for HIV, obtaining condom whenever is needed and access to sterile needles and syringes. Similar to our findings, a systematic review in 2018 indicated that there is a linkage between perceived HIV risk and uptake of preventive interventions for HIV [49]. A possible reason for not accessing HIV preventive services may be fear of the HIV test results or that they assume that they are already infected therefore there is no purpose to access or utilizing such service.

Our study found that only about 4 out of 10 PWID had correct comprehensive HIV knowledge. This level of knowledge is similar to that reported in a study done in Brazil [50] but far lower than findings reported from Vietnam [51]. Moreover, the proportion of correct comprehensive HIV knowledge was slightly lower than that of the adult general population in Tanzania [33]. The observed lower level of knowledge may be attributed to the overall low literacy level among PWID compared to the general population in Tanzania.

Our findings should be interpreted in light of the following limitations. First, the respondent-driven sampling technique was used to recruit the study participants. This being a non-probabilistic sampling technique may have introduced selection bias. However, the results were controlled for network size in our analysis to control for potential bias emanating from RDS sampling. Second, sexual practices such as condom use, injecting practices like use of sterile needles and syringes, and HIV testing, may be perceived as sensitive issues hence participants may have provided socially desired answers. Lastly, this study was done in Dar es Salaam, an urban setting with many health programming addressing HIV for key and vulnerable populations including people who inject drugs. Access to services may be overestimated in Dar es Salaam and not reflect the proportion of access in other areas of the country.

Findings from this survey provide important insights on the levels of access to different HIV preventive services among PWID. First, the levels of access to HIV testing, sterile needles and syringes, as well as access to condom were relatively high. However, our study measured a lifetime HIV testing and not the current HIV testing behaviours among PWID. Determining recent testing behaviour is critical for programming hence we recommend future studies to provide such estimate. In addition, there is a need to include the UNAIDS second and third 90’s targets to better understand the overall continuum of HIV preventive services. For programming purposes, more efforts are needed to increase HIV testing services, condom use and needle and syringe exchange programs. Secondly, HIV prevention programmes should factor in the socio-cognitive factors such as the perception of HIV risk, stigma, HIV knowledge, and violence. There is a need to tailor health education programs to increase HIV knowledge and target HIV risk perception which prevents PWID from accessing HIV preventions services. Thirdly, there is a need to address other social factors such as sexual violence among PWID.

## Supporting information

S1 Dataset Copy of the questionnaire in the original language. (XLSX)

S2 Dataset Minimal dataset. (XLSX)

S1 File Original questionnaire. (PDF)
