# Supplementary material for: Socio-cognitive factors influencing access to HIV prevention services among people who inject drugs in Dar es Salaam, Tanzania: An integrated bio-behavioural survey
Source: PLoS One. 2022 Jan 28;17(1):e0261500. doi: 10.1371/journal.pone.0261500 (PMC8797198; doi:10.1371/journal.pone.0261500)
Supplement: S1 File — (PDF) [file pone.0261500.s003.pdf]

Dar BSS 2017 - PWID_30Oct2017

Scan barcode

___

First, I would like to ask you a few questions on your background, including information on your age, education, jobs and income.

Sex

- Male
- Female

How old are you?

(In completed years)

___

How many years of education have you completed up to now?

- Never went to school
- Did not complete primary
- Completed primary
- Did not complete secondary
- Completed secondary
- Higher than secondary education
- No response

What is your current marital status?

Do not read out the possible answers. Mark only one response.

- Currently married/living with a partner
- Separated, divorced, or widowed
- Never married
- No response

How long have you lived here (Dar)?

If number of years in Dar is unknown, ask for an estimate. If less than one year, enter 0. If 1 1/2 round up to 2. If born and raised in Dar, enter 96. If participant refuses to respond, enter 98.

____

Currently, with whom are you living?

Read out the possible answers. Mark only one response.

- Alone
- With wife/girlfriend
- With husband/boyfriend
- With family
- With friends
- No fixed address (unsettled)
- No response
- Other

Specify other.

____

How much income did you earn in the past month?

Answer in Tanzanian shillings. If exact amount is not known, ask for an estimate.

____

How do you earn money?

Do not read out the possible answers. Mark all mentioned.

- [ ] Emplyed by government/parastatal
- [ ] Employed by private company
- [ ] Self-employed
- [ ] Student
- [ ] Petty trading
- [ ] Illegal activities
- [ ] Currently unemployed
- [ ] No response
- [ ] Other

Specify other.

Did you participate in a study where you received a GREEN coupon?

Refers to ongoing MSM study - not studies done in previous years.

- ○ Yes
- ○ No
- ○ Don't remember
- ○ No response

Did you participate in a study where you received a RED coupon?

Refers to ongoing FSW study - not studies done in previous years.

- ○ Yes
- ○ No
- ○ Don't remember
- ○ No response

Have you participated in any studies like this one where you received a coupon about 4 years ago, in 2013?

Refers to study done in 2013.

- Yes
- No
- Don't remember
- No response

Now I would like to ask you some questions about other PWID that you may know, including the person who recruited you into this study.

How many PWID do you know personally?

Only count those who live in Dar, are 15 years and above, you know their name, you know who they are and they know you. If exact number is unknown, ask for an estimate.

____

How many of these (repeat the number given in prior question) PWID have you seen during the past one month?

If exact number of PWID is unknown, ask for an estimate.

____

Would you have given a coupon to the same person who gave this coupon to you?

- Yes
- No
- No response

What is the primary reason you decided to accept a coupon and enroll in the study?

Do not read responses. Mark only one response.

- For incentive
- For STI/HIV test results
- Peer influence
- Study seems interesting/useful
- I wasn't busy
- Don't know
- No response
- Other

Specify other.

Which of the following best describes your relationship to the person who recruited you?

Read responses. Mark only one response.

- A stranger, someone you met for the first time
- Someone you know, but not closely
- A close friend, someone you know very well
- A sexual partner
- A family member or relation
- A drug dealer
- Someone you inject with
- No response

How often do you see the person who referred you to this study?

Do not read responses. Mark only one response.

- Every day
- Once per week
- Once per month
- Less than once per month
- No response

About how long have you known the person who referred you to this study?

Do not read responses. Mark only one response.

- Less than 6 months
- 6 months to 1 year
- 1-2 years
- More than 2 years
- No response

Did you ever receive this object?

Show object to participant.

- Yes
- No
- No response

When did you receive this special object?

- 20-27 October 2017
- Other time
- No response

What color was the object you received?

Individual could have received more than one object. Mark all that apply.

- Pink
- Green
- Purple
- Don't remember

Now I would like to ask you some questions drug use, with and without a needle. These are very personal matters but they are very important for providing health services. Please remember that the answers to your questions are confidential and completely private.

In the past one month, how often did you have a drink containing alcohol?

Do not read responses. Mark one response only.

- Never
- Once a month or less
- 2-4 times a month
- 2-3 times a week
- 4 or more times a week
- Don't remember
- No response

How many drinks containing alcohol do you have on a typical day when you are drinking?

Do not read responses. Mark one response only.

- 1 or 2
- 3 or 4
- 5 or 6
- 7, 8 or 9
- 10 or more
- Don't remember
- No response

How old were you when you first injected drugs?

If exact age is unknown, ask for an estimate.

____

Who was the person who introduced you to injecting?

- Husband/wife
- Boyfriend/girlfriend
- Friend
- Neighbor
- Family member
- Drug seller
- Other drug user
- Don't remember
- No response
- Other

Specify other.

____

Have you taken any non-injected drugs other than alcohol in the last three months?

- Yes
- No
- Don't know
- No response

What types of non-injected drugs have you used in the past three months?

Do not read responses. Probe and select all that apply.

- Smoked hashish/marijuana
- Smoked crack cocaine
- Smoked heroin
- Inhaled cocaine
- Mixed cocktail
- Chase the dragon
- Sniffed petrol, glue
- Valium
- Pain killers (prescription drugs)
- Other

Specify other.

Which types of drugs have you injected in the past three months?

Do not read responses. Probe and select all that apply.

- ☐ Brown heroin
- ☐ White heroin
- ☐ Opium
- ☐ Amphetamines
- ☐ Prescription drugs
- ☐ Other

Specify other.

____

During the past one month, on average, how often did you inject drugs?

Do not read responses. Mark one response only.

- ○ Once a month or less
- ○ Several times a month, but less than once a week
- ○ Once a week
- ○ Several times a week, but not every day
- ○ Once a day
- ○ Several times a day
- ○ Don't remember

During the past one month, where did you most often get your needle/syringe?

Do not read responses. Mark one response only.

- Pharmacy
- Health establishment
- Drug seller
- Fellow drug user
- Outreach health worker
- Peer educator
- Drop-in center
- Private home known to have clean needles available
- No response
- Other

Specify other.

Can you get a clean needle and syringe any time you need one?

Do not read responses. Mark one response only.

- Yes
- No
- Never tried
- No response

What things make it difficult for you to access clean needles/syringes?

Read responses. Multiple options possible.

- [ ] Needles/syringes too expensive
- [ ] Vendor/needle seller closed or not around
- [ ] Preferred size not available
- [ ] Vendor ran out/stock out
- [ ] Vendor too far away
- [ ] Do not know where to get
- [ ] No need
- [ ] Retailers refuse to sell to me
- [ ] No response
- [ ] Other

Specify other.

____

Last time you were able to get a clean needle, where did you get it?

- ( ) Pharmacy
- ( ) Health establishment
- ( ) Drug seller
- ( ) Fellow drug user
- ( ) Outreach health worker
- ( ) Peer educator
- ( ) Drop-in center
- ( ) Private home known to have clean needles available
- ( ) No response

During the past one month, did you inject blood from someone who had taken drugs? (Flashblood)

- Yes
- No
- Don't know/don't remember
- No response

Now I would like to ask you some questions about sharing needles. Sharing means using the same needle and/or syringe as someone else to inject drugs.

Have you ever shared a needle with someone else when you injected?

- Yes
- No
- Don't know/don't remember
- No response

In the past one month, when you injected, did you use a needle previously used by someone else?

- Yes
- No
- Don't know/don't remember
- No response

During the past one month, when you injected, how often did you use needles/syringes that had previously been used by someone else?

- Always
- Most of the time
- Occasionally
- No response

During the past one month, how often did you clean the syringe and needle that had previously been used by someone else before you used it again?

- Always
- Most of the time
- Occasionally
- Never
- Don't remember
- No response

If cleaned, how did you usually clean the syringe and needle?

Read list. Mark all that apply.

- Cold water
- Hot water
- Bleach
- Soap
- Alcohol
- Other

Specify other.

During the past one month, have you shared needles/syringes with:

Read list, select all mentioned.

- Wife/girlfriend
- Husband/boyfriend
- Sex worker
- Someone who paid you for sex
- Other sexual partner
- Injecting drug user
- Shooting gallery person
- No response
- Other

Specify other.

Now I would like to ask you some questions about the last time you injected drugs.

The last time you injected, what drug did you use?

Do not read responses. Mark one response only.

- Brown heroin
- White heroin
- Opium
- Amphetamines
- Prescription drugs
- Other

Specify other.

The last time you injected, how much did you spend on the drugs?

Answer in Tanzanian shillings. If exact amount is unknown, ask for an estimate. If they give a range, provide the average.

The last time you injected, did you use a new sterile needle, a needle with an intact seal that had never been used before, not even by you?

- Yes
- No
- Don't know/don't remember
- No response

The last time you injected, how many other injectors shared the same needle/syringe?

If exact number of injectors is unknown, ask for an estimate. Number should not include interviewee.

Now I would like to ask you a few questions about the last time you shared a needle/syringe. This is not necessarily the last time you injected.

The last time you shared needles/syringes with other users, what was the reason?

- Needles/syringes too expensive
- Prefer to share with friend
- Other injector wanted me to
- Did not have enough money to inject alone
- Cannot inject myself
- Syringes/needles not available
- No response
- Other

Specify other.

The last time you shared needles/syringes with other users, was the needle/syringe cleaned between users?

- Yes
- No
- Don't know/don't remember
- No response

The last time you shared needles/syringes with other users, what did you use to clean the needle/syringe?

Read list. Mark all that apply.

- Cold water
- Hot water
- Bleach
- Soap
- Alcohol
- Other

Specify other.

Now I would like to ask you some questions about your sexual history, your sex partners, and your use of condoms. These are very personal matters but they are very important for providing health services. Please remember that your answers will remain completely confidential. Let's first talk about your non-paying sexual partners.

Have you ever had sex with a man or woman where no payment was involved?

- Yes
- No
- No response

In the past one month, have you had sex with a man or woman where no payment was involved?

- Yes
- No
- No response

Was this man/woman your boyfriend/girlfriend/husband/wife or other steady partner?

- Yes
- No
- No response

In the past one month, how many partners have you had vaginal or anal sex with where no payment was involved?

If exact number of partners is unknown, ask for an estimate.

____

Of all times you have vaginal or anal sex with a non-paying male or female partner in the last month, how frequently did you use a condom?

- Always
- Most of the time
- Occasionally
- Never
- Didn't have sex past month
- Don't remember
- No response

The last time you had vaginal or anal sex with a non-paying male or female partner, did you use a condom?

This could be before the past one month.

- Yes
- No
- Don't remember
- No response

Now I would like to ask you some questions about people you pay for sex. These could be male or female partners you give money or gifts to in exchange for sex.

Have you ever paid any woman or man to have vaginal or anal sex with you?

- Yes
- No
- No response

In the past one month, have you paid any woman or man to have vaginal or anal sex with you?

- Yes
- No
- No response

In the past one month, how many different women have you paid to have sex with you?

If the exact number of women is unknown, ask for an estimate. If the answer is none, write 0.

____

In the past one month, how many different men have you paid to have sex with you?

If the exact number is unknown, ask for an estimate. If the answer is none, write 0.

____

In the past one month, how many times did you pay for sex?

If the exact number is unknown, ask for an estimate.

____

Of all times you paid someone to have sexual intercourse with you in the last month, how frequently did you use a condom?

- Always
- Most of the time
- Occasionally
- Never
- Didn't have sex past month
- Don't remember
- No response

The last time you paid someone for vaginal or anal sex, did you use a condom?

This could be before the past one month.

- Yes
- No
- Don't remember
- No response

Now I will ask you some questions about people who pay you to have sex with them. These could be friends or people you just met who give you money or gifts to have sex with them.

Has any woman or man ever paid you to have vaginal or anal sex with them?

- Yes
- No
- No response

In the past one month, has any woman or man paid you to have vaginal or anal sex with them?

- Yes
- No
- No response

In the past one month, how many different women have paid to have vaginal or anal sex with you?

If the exact number of women is unknown, ask for an estimate.

____

In the past one month, how many different men have paid to have sex with you?

If the exact number is unknown, ask for an estimate.

____

Of all times someone paid you for vaginal or anal sex in the past one month, how frequently did you use a condom?

- Always
- Most of the time
- Occasionally
- Never
- Didn't have sex past month
- Don't remember
- No response

The last time a man or woman paid you for vaginal or anal sex, did you use a condom?

This could be before the past one month.

- Yes
- No
- Don't remember
- No response

Now I will ask you some questions on condom use. These are very personal matters but they are very important for providing health services.

Have you ever used a male condom?

If respondent is a woman, emphasize that it is her partner wearing the condom

- Yes
- No
- Don't remember
- No response

If you had vaginal or anal sex during the past one month, how often did you use a male condom?

Do not read responses.

- Always
- Most of the time
- Occasionally
- Never
- Didn't have sex past month
- Don't remember
- No response

Can you obtain a male condom every time you need one?

- Yes
- No
- Never needed one
- Don't know
- No response

Why can't you get a male condom every time you need one?

Multiple answers possible. Do not read outloud.

- [ ] Costs too much
- [ ] Shop too far away
- [ ] Shops closed
- [ ] Pharmacy too far away
- [ ] Pharmacy closed
- [ ] Embarassed to buy condom
- [ ] Don't know where to obtain
- [ ] Things happen too fast
- [ ] Don't need condom
- [ ] Don't know
- [ ] No response
- [ ] Other

Specify other.

Which places or persons have you obtained male condoms from in the last one month?

Do not read responses. Probe and select all that apply.

- [ ] Shop
- [ ] Pharmacy
- [ ] Health facility
- [ ] Bar/guesthouse/ hotel
- [ ] Friends
- [ ] Taxi drivers
- [ ] Saloon
- [ ] NGO
- [ ] Public office
- [ ] Peer educator
- [ ] Did not buy male condom in the last month
- [ ] Don't remember
- [ ] No response
- [ ] Other

Specify other.

Have you ever used a female condom?

- ○ Yes
- ○ No
- ○ Don't remember
- ○ No response

Where or from whom did you obtain your last female condom?

- Shop
- Pharmacy
- Health facility
- Bar/guesthouse/ hotel
- Friends
- Taxi drivers
- Saloon
- NGO
- Public office
- Peer educator
- Don't remember
- No response
- Other

Specify other.

Now I will ask you some questions on violence and history of incarceration. These questions are personal and may make you uncomfortable. If they do so, you may choose to not answer the question.

In the past 12 months, were you ever beaten?

- Yes
- No
- Don't remember
- No response

Who was the person (or people) who physically beat you?

Multiple answers possible. Do not read outloud. Select all that apply.

- ☐ Police
- ☐ Drug dealer
- ☐ Husband/boyfriend
- ☐ Wife/girlfriend
- ☐ Friends
- ☐ Family member
- ☐ Unknown person
- ☐ Do not remember
- ☐ No response
- ☐ Other

Specify other.

____

In the past 12 months, were you ever forced to have sex?

- ◯ Yes
- ◯ No
- ◯ Don't remember
- ◯ No response

Who was the person (or people) who forced you to have sex?

Multiple answers possible. Do not read outloud. Select all that apply.

- [ ] Police
- [ ] Drug dealer
- [ ] Husband/boyfriend
- [ ] Wife/girlfriend
- [ ] Friends
- [ ] Family member
- [ ] Unknown person
- [ ] Do not remember
- [ ] No response
- [ ] Other

Specify other.

____

During the past 12 months, have you been arrested?

- ( ) Yes
- ( ) No
- ( ) Don't remember
- ( ) No response

[If yes] What were you arrested for?

Multiple answers possible. Do not read outloud. Select all that apply.

- Drug use
- Aggravated assault
- Theft
- Selling sex
- Loitering
- Selling drugs
- Don't know/remember
- No response
- Other

Specify other.

Now I would like to ask you some questions about stigma that may affect you because you inject drugs.

Does anyone in your family know that you inject drugs ?

- Yes
- No
- Don't know
- No response

Please answer yes or no to the following statements that refer to your experiences as a PWID in your adult life (>15 years old).

I have experienced name calling, teasing and insults.

- Yes
- No
- Don't know/don't remember
- No response

I have been excluded from a social gathering.

- Yes
- No
- Don't know/don't remember
- No response

I have been gossiped about.

- Yes
- No
- Don't know/don't remember
- No response

Other people have lost respect for me.

- Yes
- No
- Don't know/don't remember
- No response

I have been abandoned by my loved ones.

- Yes
- No
- Don't know/don't remember
- No response

Now I will read some statements about HIV/AIDS. Some of them are true and some are not true. These are general statements and do not refer to your own experience or behavior. Please tell me whether you agree or disagree with each of the statements.

Can the risk of HIV transmission be reduced by having sex with only one uninfected partner who has no other partners?

- Yes
- No
- Don't know
- No response

Can a person get HIV from mosquito bites?

- Yes
- No
- Don't know
- No response

Can a person reduce their risk of getting HIV by using a condom every time they have sex?

- Yes
- No
- Don't know
- No response

Can a person get HIV by sharing food with someone who has HIV?

- Yes
- No
- Don't know
- No response

Can a healthy looking person have HIV?

- Yes
- No
- Don't know
- No response

Sharing needles when injecting drugs will increase the risk of HIV infection.

- Yes
- No
- Don't know
- No response

Cleaning needles and syringes between injections reduces the risk of HIV.

- Yes
- No
- Don't know
- No response

Have you ever heard of a medication you can take that is like ARVs but you take it to prevent getting HIV?

- Yes
- No
- No response

If there were a drug you could take everyday to prevent HIV infection would you want to take it?

- Yes
- No
- Don't know
- No response

[If no] Why not?

- I worry about someone finding out and thinking I am HIV positive.
- I don't like taking drugs.
- I don't think I am at risk for HIV.
- I am not able to access services easily.
- I worry about side effects from drugs.

Now I will ask some questions about stigma related to HIV/AIDS. Please tell me whether you agree or disagree with each of the statements.

People with HIV/AIDS should be ashamed of themselves

- Agree
- Disagree
- Don't know

I would feel ashamed if someone in my family had HIV/AIDS

- Agree
- Disagree
- Don't know

I would feel ashamed if I were infected with HIV/AIDS

- Agree
- Disagree
- Don't know

People with HIV/AIDS are promiscuous

- Agree
- Disagree
- Don't know

It is PWID who spread HIV in the community

- Agree
- Disagree
- Don't know

HIV/AIDS is brought as a punishment for bad behavior

- Agree
- Disagree
- Don't know

Now I will ask you about how you see your risk for HIV.

With your current behaviors, how do you think about your risk of HIV infection?

- High risk
- Medium risk
- Low risk
- No risk
- Don't know
- No response

If you feel you are at risk, why do you feel that you are at risk for HIV infection?

Multiple responses possible, but do not read choices aloud.

- I often change sex partners.
- I don't always use a condom.
- I use drugs.
- I inject drugs.
- I share needles.
- I have sex with IDUs.
- Don't know
- No response
- Other

Specify other.

If you feel you are not at risk, why do you feel that you are not at risk for HIV infection?

Multiple responses possible, but do not read choices aloud.

- [ ] I am faithful
- [ ] I always use condoms
- [ ] I'm convinced my partner is clean
- [ ] I don't have anal sex
- [ ] I never have sex with sex workers
- [ ] Don't know
- [ ] No response
- [ ] Other

Specify other.

____

Now I want to ask you about HIV testing and your use of other HIV health services.

Do you know of a place where people can go to have a confidential test to find out if they are infected with HIV?

Confidential means that nobody will know the test result unless you want them to know

- ○ Yes
- ○ No
- ○ No response

Have you ever had an HIV test?

- ○ Yes
- ○ No
- ○ No response

When did you last get an HIV test for which you received the results?

- In the past year
- Over one year ago
- Never
- Don't remember
- No response

Where did you get your last HIV test for which you received the results?

- Health facility including hospital
- Community
- Self-test
- Research projects
- No response

What was the result of your last HIV test for which you got the results?

- Positive
- Negative
- Not comfortable saying

Have you enrolled in HIV care and treatment services?

- Yes
- No
- No response

[If yes] How were you linked to services?

- Went on my own with referral slip
- Escorted by peer from NGO
- Escorted by facility staff to CTC
- Escorted by CBHS

[If yes] How long was it from your first HIV+ test before you enrolled in HIV care and treatment services?

- Same day
- Within one week
- Within two weeks
- Longer than two weeks
- Don't remember

[If yes] Are you currently on ART?

- Yes
- No
- No response

[If yes] How long have you been on ART?

- Less than three months
- Three to five months
- Six months or longer
- Don't know/don't remember
- No response

Are you currently pregnant or breastfeeding?

- Yes
- No
- No response

[If no] Why not?

- Was not prescribed ART
- Opted out of ART/did not want to take ART
- Other

Specify other.

____

Why have you not enrolled in care and treatment services?

- Don't think I need to enroll now
- Worried about side effects of ARVs
- Worried about being badly treated by HCWs
- Worried someone they know might see them at CTC
- Went to alternative treatment

Why have you never chosen to get an HIV test?

Probe and select all that apply.

- [ ] Didn't know where to go
- [ ] Don't feel at risk
- [ ] Concerned about confidentiality
- [ ] Negative attitude of health care workers
- [ ] Cost
- [ ] Distance
- [ ] Fear of knowing status
- [ ] Not important for me
- [ ] Don't know
- [ ] No response
- [ ] Other

Specify other.

Have you ever attended HIV counseling with your permanent/steady partner?

- Yes
- No
- Don't remember
- No response

Have you talked to your partner/boyfriend/husband about the results of your HIV tests?

- Yes
- No
- Don't remember
- No response

In this last section, I will ask you some questions about the health services you have accessed and your experience with those services.

The last time you had a health concern, where did you go?

- Pharmacy
- Public health facility/hospital
- Private health facility/hospital
- Outreach
- Peer educator/NGO
- Treated myself at home
- Alternative treatment
- Did not do anything/go anywhere
- Don't remember
- No response

Why did you not seek services for your health concern?

Probe and select all that apply.

- Not enough money
- Did not think I would be treated well by provider
- Worried about seeing someone I know
- Thought health problem would go away by itself
- Don't remember
- No response
- Other

Specify other.

The last time you visited a health facility, what services did you receive?

If they have received more than 1 service, mark all that apply

- ☐ Received condoms
- ☐ Information on STI or HIV transmission or prevention
- ☐ Counseling from a professional/VCT counselor
- ☐ An HIV Test
- ☐ General OPD services
- ☐ Family planning
- ☐ ANC/PMTCT
- ☐ Care and treatment
- ☐ Don't remember
- ☐ No response
- ☐ Other

Specify other.

____

Based on the way you were treated by the facility staff, would you return to that facility for services?

- ○ Yes
- ○ No
- ○ Don't know
- ○ No response

[If not] Which of these did you experience that makes you not want to return to that facility?

Read out the responses, mark all that apply

- Health workers spoke unkindly to you
- Health workers gossiped about you to other health workers/clients
- Health workers shared information about you and your drug use behaviors with other health workers/clients
- Health workers did not take time to explain medications or procedures to you
- Health workers were physically abusive to you
- Health workers avoided physical contact with you

Have you ever received Opioid Substitution Therapy?

- Yes
- No
- No response

[If yes] For how long were you in Opioid Substitution therapy?

- Less than six months
- More than six months

Have you been in contact with any health peer educator in the community in the last 12 months?

- Yes
- No
- Is a peer educator
- Don't know
- No response

How many times have you been in contact with a peer educator in the last 12 months?

- One time only
- Two times
- Three times
- Four times
- Five or more times
- Don't remember
- No response

What services or information did you receive from the peer educator?

Read the answer choices aloud. Mark the service that applies; if they have received more than 1 service mark all that apply

- General STI or HIV transmission or prevention information
- Condoms
- HIV test in your home
- Lubrication
- Referral for STI treatment
- Referral for VCT
- Referral for PMTCT or family planning
- Referral for TB screening
- Bleach kit
- Clean needles
- Don't remember
- No response
- Other

Specify other.

Did you feel that the peer educators were non-judgmental?

- Yes
- No
- Don't remember
- No response

The last time you received services in the community from a peer educator, which NGO was supporting those services?

- Sauti
- MDH
- Passada
- MDM
- Never received services in the community from a peer educator
- Don't know
- Other

Specify other.

____

Have you been in contact with any community home-based care providers in the last 12 months?

- Yes
- No
- Don't remember
- No response

READ ALOUD: We have come to the end of the interview. Thank you very much for your kind cooperation and spending your valuable time with me.

Number of recruitment coupons given:

- One
- Two
- Three

Scan first recruitment coupon.

Scan first recruitment coupon.

Scan second recruitment coupon

Scan first recruitment coupon.

Scan second recruitment coupon

Scan third recruitment coupon.

Mark on the Laboratory Requisition Form and in the Laboratory Tracking Logbook whether a viral load measurement is required.
